# Systemic allergic contact dermatitis to palladium, platinum, and titanium: mechanisms, clinical manifestations, prevalence, and therapeutic approaches

**DOI:** 10.1002/mco2.386

**Published:** 2023-10-21

**Authors:** Ali Sadrolvaezin, Arezou Pezhman, Iman Zare, Shima Zahed Nasab, Sajad Chamani, Ali Naghizadeh, Ebrahim Mostafavi

**Affiliations:** ^1^ Medical Toxicology and Drug Abuse Research Center Birjand University of Medical Sciences Birjand Iran; ^2^ School of Medicine Zahedan Azad University of Medical Sciences Zahedan Iran; ^3^ Research and Development Department Sina Medical Biochemistry Technologies Co. Ltd. Shiraz Iran; ^4^ Department of Life Science Engineering Faculty of New Sciences and Technologies University of Tehran Tehran Iran; ^5^ Stanford Cardiovascular Institute Stanford University School of Medicine Stanford California USA; ^6^ Department of Medicine Stanford University School of Medicine Stanford California USA

**Keywords:** allergic contact dermatitis, clinical manifestations, heavy metals, prevalence, systemic contact dermatitis

## Abstract

Contact dermatitis (CD) is an inflammatory skin disease of eczema that is elicited by chemicals or metal ions that have toxic effects without eliciting a T‐cell response (contact elicitation) or by small reactive chemicals that modify proteins and induce innate and adaptive immune responses (contact allergens). The clinical condition is characterized by localized skin rash, pruritus, redness, swelling, and lesions, which are mainly detected by patch tests and lymphocyte stimulation. Heavy metals such as palladium (Pd), platinum (Pt), and titanium (Ti) are ubiquitous in our environment. These heavy metals have shown CD effects as allergic agents. Immunological responses result from the interaction of cytokines and T cells. Occupational metal CD accounts for most cases of work‐related cutaneous disorders. In this systematic review, the allergic effects of heavy metals, including Pd, Pt, and Ti, and the mechanisms, clinical manifestations, prevalence, and therapeutic approaches are discussed in detail. Furthermore, the therapeutic approaches introduced to treat CD, including corticosteroids, topical calcineurin inhibitors, systemic immunosuppressive agents, phototherapy, and antihistamines, can be effective in the treatment of these diseases in the future. Ultimately, the insights identified could lead to improved therapeutic and diagnostic pathways.

## Graphical abstract

Heavy metals such as titanium, palladium, and platinum cause allergic reactions (contact dermatitis) after exposure to the skin. Immunological responses result from the interaction of cytokines (secreted by keratinocytes) and T cells, which cause skin inflammation.

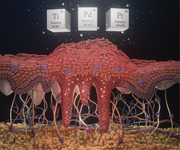

## 1 INTRODUCTION

The term heavy metals generally refers to metals and metalloids with a density greater than 5 g/cm^3^ and an atomic number greater than 20, which cause environmental pollution and toxicity. Nowadays, experts believe that the term heavy metal should be revised and a more accurate definition and name should be substituted for this group of elements.^1^ Various heavy metals, such as mercury, lead, arsenic, palladium (Pd), platinum (Pt), etc., enter the body through contaminated food and contaminated water, breathing, and skin contact and then exert their toxic effects through various mechanisms.^2^ Reaction with the body's chemical compounds (e.g., chlorine and oxygen), replacing and competition with the body's essential minerals, and disruption of metabolic reactions, enzyme function, antioxidants, and hormones, as well as neurotransmitter disorders, DNA damage, and hypersensitivity reactions, are some of the mechanisms by which heavy metals exert their toxicity.^3^ These mechanisms induce heavy metal complications such as cardiovascular disease, kidney failure, infertility, neurological disorders, cancer, diabetes, developmental disorders, and allergy.^4^ Allergy is an overreaction of the immune system to harmless antigens called allergens, while normal people are not sensitive to this group of antigens. Allergic disorders affect about 25% of the world's population and are increasing in developed countries.^5^ There are different types of allergic disorders, such as atopic dermatitis (also known as atopic eczema), allergic rhinitis (also known as hay fever), allergic (or atopic) asthma, food allergies, anaphylaxis, and contact dermatitis (CD) (an inflammatory skin disease).^6^, ^7^, ^8^, ^9^

Skin problems related to chemical contact are constantly growing environmental and occupational health problems. Two main types of CD have been described according to the pathophysiological mechanism involved: irritant CD (ICD) and allergic CD (ACD).^10^, ^11^, ^12^, ^13^, ^14^, ^15^, ^16^ ICD is the most common occupational skin disease, accounting for 80% of all CD cases. It can occur after a single exposure to an irritant or toxic substance (e.g., abrasives, cleaning, oxidizing, and reducing agents) and is characterized by skin damage caused by a direct, local cytotoxic effect on skin cells.^17^, ^18^ ACD accounts for 20% of CD cases. It is an adverse inflammatory skin reaction after direct skin contact with a specific exogenous allergen.^19^, ^20^, ^21^ After exposure to several sensitizing agents, individuals develop clinical manifestations often characterized by severe itching, burning, and pain with marked erythema and edema.^17^ The induced CD is a non‐specific skin response to direct chemical damage to the skin and/or by releasing inflammatory mediators, whereas ACD is a delayed (type IV) hypersensitivity reaction to allergens involving immune responses (due to interaction of T cells and cytokines).^22^, ^23^ In differentiating these two diseases, it is emphasized that there is no immune reaction in CD stimulus. No prior contact with any substance (sensitization) is required, and most people exposed to such a (usually aggressive) substance will react similarly.^24^, ^25^ Metal allergy may lead to ACD. Electrophilic metals can ionize and react with proteins, thus forming complexes that can be recognized by dendritic cells (DCs), allowing sensitization.^26^ Table 1 shows the “classic” clinical forms of CD.^27^

**TABLE 1:** Clinical forms of contact dermatitis (CD) and relevant mechanisms.^27^

Form of dermatitis	Description
ICD	Lesions restricted to the site of toxin exposure Demarcated in the acute stage A broad spectrum of erythema to necrosis Manifestations are highly dependent on severity and toxin Non‐distribution
ACD	Specific immunological sensitivity to contact allergens The area and configuration of the dermatitis are generally unspecified indicating the eliciting factor Diffusion reactions, movement away from the initial site of exposure, are common
Airborne ACD	Dermatitis in exposed areas of the body caused by allergens in the air (wall paint, plants, etc.)
Photo‐CD	Photoallergies require prior sensitization Occurs primarily in areas exposed to light Substances that are toxic when exposed to light (e.g., furocoumarin) cause irritant dermatitis in the absence of sensitization
Asteatotic dermatitis	Dry and cracked skin with red cracks, especially in old or damaged skin (improper care, excessive washing)
“Dry” chronic CD	On fingers and hands due to occupational dermatosis in dentists and gardeners
Dyshidrotic dermatitis or pompholyx hematogenous CD	Special clinical form of CD (differential diagnosis, special form of atopic dermatitis)
Transfer CD	The allergen is transferred to other areas of skin without primary contact with the allergen (e.g., eyelids)
Connubial CD	Facial CD following para‐phenylenediamine sensitization due to spouse's dyed hair

Abbreviations: ACD, allergic CD; ICD, irritant CD.

*Source*: Reproduced from Ref.^27^

In this review, Pd, Pt, and titanium (Ti) are discussed as three main metals. Pd, Pt, and Ti, with atomic numbers 46, 78, and 22, respectively are precious metals that are widely used in industrial and biomedical applications. Due to the increasing use of these metals, especially their use in making prostheses, jewelry, and anticancer drugs that are directly related to the human body, more attention should be paid to the issue of allergic reactions. The molecular mechanisms related to CD are briefly described. Meanwhile, the molecular mechanisms of heavy metals and the allergic effects of CD are presented. On the other hand, the clinical manifestations of all three heavy metals Pd, Pt, and Ti were explored in depth. Furthermore, various therapeutic approaches, including corticosteroids, topical calcineurin inhibitors, systemic immunosuppressive agents, phototherapy, and antihistamines, have been introduced. Finally, therapeutic perspectives for quality improvement in the diagnosis and treatment of ACD diseases are presented.

## 2 CONTACT DERMATITIS AND RELATED MECHANISMS

CD (a type of eczema) is an inflammatory skin disease caused by skin contact with chemical or physical agents. There are two main types of CD. The more common type is ICD (80% of CD cases), which is a non‐immunological response of the skin to toxic or irritating chemicals. ICD may appear acutely or chronically. Any substance that causes acute CD can cause chronic CD. Another type is ACD (20% of CD cases), which is a T‐cell‐mediated immune response that follows contact with allergens such as nickel (Ni) (the most common agent), poison ivy, and heavy metals.^28^, ^29^, ^30^, ^31^, ^32^ Metal ions act as haptens, which are not immunogenic per se, and bind to cellular and extracellular protein structures called carriers.^33^, ^34^, ^35^ The carrier hapten complex then binds with DC receptors to be presented to T lymphocytes. Likewise, haptens also induce keratinocytes to express intercellular adhesion molecule‐1 and proinflammatory cytokines (i.e., interleukin [IL]‐1α, IL‐1β, tumor necrosis factor‐alpha [TNF‐α], IL‐6) and chemokines (e.g., IP‐10, MCP‐1, RANTES, CCL18). In the early stage, stimulatory innate immune signals are required to activate DCs and their migration to cutaneous lymph nodes, where DCs activate T lymphocytes by presenting haptens to these cells. When the next contact occurs, antigen‐presenting cells (APCs) present the antigen to memory CD8^+^ lymphocytes, and memory CD8^+^ lymphocytes then activate cytotoxic T lymphocytes (CTLs). CTLs release interferon‐gamma (IFN‐γ), which stimulates the expression of Fas molecules on keratinocytes. Fas ligands are expressed by CTLs and Fas ligand/receptor interaction leads to keratinocytes apoptosis. Furthermore, IFN‐γ stimulates mastocytes and basophils to release vasodilators, lytic mediators, and chemokines that promote immune cell infiltration and potentiate allergic responses. In addition to IFN‐γ, CD8^+^ cells release perforin and granzyme to exert their cytotoxic effects. Despite the cytotoxic effect of perforin and granzyme, they are not essential for the induction of contact allergy.^30^, ^36^, ^37^, ^38^, ^39^, ^40^, ^41^

## 3 MECHANISMS OF ALLERGIC REACTIONS CAUSED BY HEAVY METALS

**FIGURE 1:**
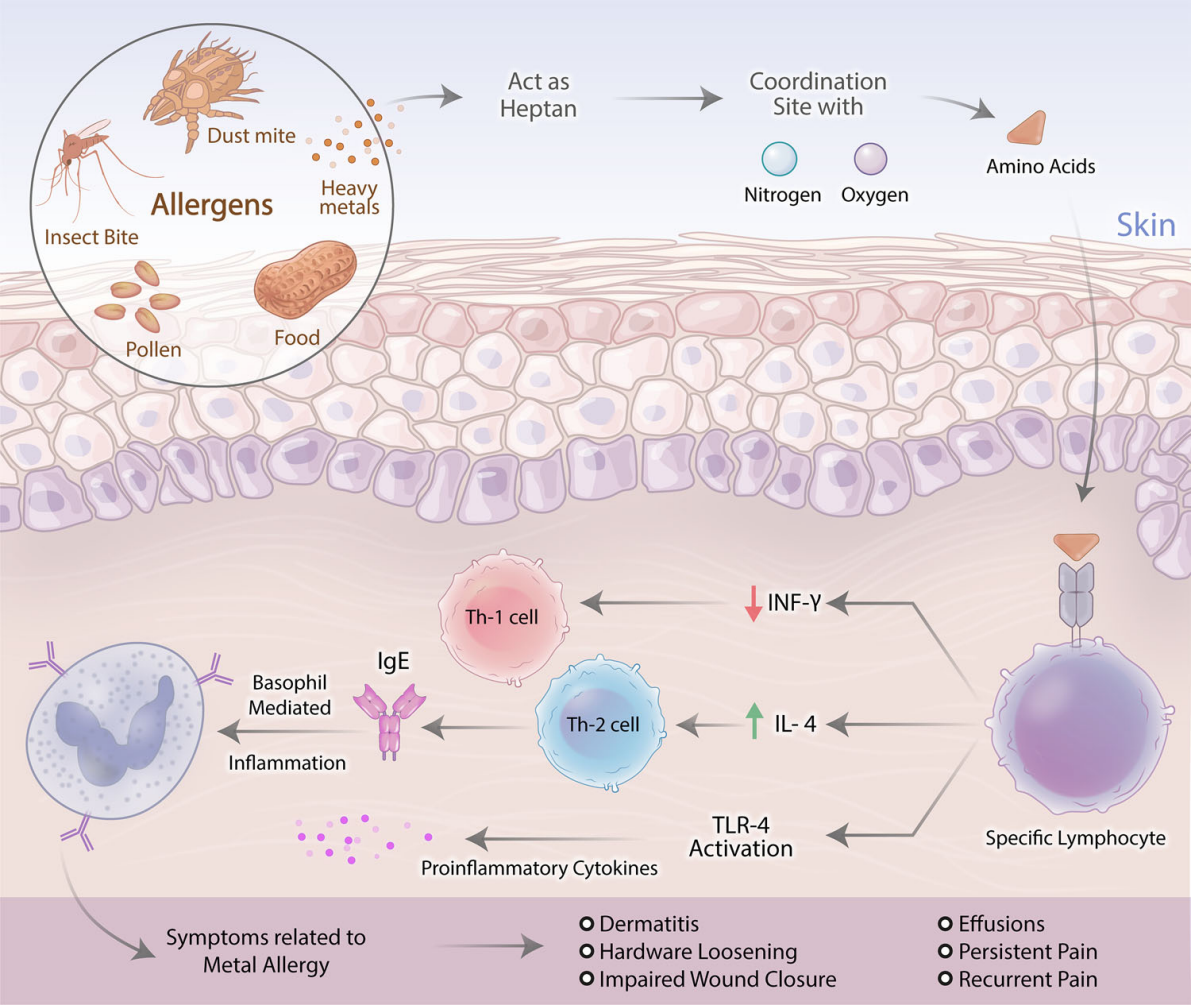
Mechanism of allergic reaction caused by heavy metals. Skin lesions are caused by immune reactions and sensitivity to some substances such as dust mite, heavy metals, food, insect bite, and pollen. This allergens acts as heptan that induces coordination sites with electron‐rich ligands like oxygen or nitrogen. It may also involve specific amino acids in the T‐cell receptor and MHC molecule, and changes in these amino acids may be related to the degree and type of hypersensitivity. Heavy metals suppress the development of Th‐1 by inhibiting the expression of interferon‐gamma (IFN‐γ) and promote the development of Th‐2 by increasing the expression of interleukin‐4 (IL‐4) and increasing the production of immunoglobulin E (IgE).

ICD lesions occur after exposure to a single substance (even at small concentrations), and the effects may accumulate due to repeated exposure, usually resulting in chronic skin damage and lesions.^42^, ^43^, ^44^ ICD may present as acute or chronic lesions. Many substances related to CD are triggers due to their irritant or toxic effects, for example, chemical agents, physical agents, plants, phototoxic agents, airborne irritants, etc.^20^, ^22^, ^24^, ^45^ Chronic ICD may be caused by any substance that causes acute ICD, which in small concentrations can produce effects that cause chronic skin damage (even water, in cases of frequent hand washing, working in water, showering, bathing, etc.).^24^, ^46^, ^47^ On the other hand, in ACD, skin lesions are caused by immune reactions and sensitivity to some substances (allergens) through T cells, which occurs through the sensitization phase (afferent) and the excitation phase (efferent) (Figure 1).^48^ The results of the patch test (PT), which is used to investigate and prove a delayed hypersensitivity reaction (type IV), are the most important diagnostic indicator in differentiating between stimulation and ACD.^24^, ^45^ Various substances can be allergens, especially metal salts that interact weakly with skin proteins and thus form complexes. Contact allergens are low‐molecular‐weight chemicals called haptens that are not immunogenic by themselves and require binding to epidermal proteins.^34^

During subsequent contact with the same hapten, in the dermis, APCs present the neoantigen to memory CD8^+^ lymphocytes, which develop CTL function. They bind to keratinocytes that express pMHC I and secrete IFN‐γ.^30^ IFN‐γ (secreted from CTLs and activated macrophages) stimulates the expression of Fas molecules on keratinocytes. In turn, IFN‐γ binds to FasL expressed by CTLs, leading to apoptosis.^30^ In the final step, IFN‐γ stimulates mast cells and basophils to release lytic mediators. It also stimulates vasodilators (such as histamine) and chemokines to allow further cell infiltration and promote an inflammatory response.^34^, ^41^

## 4 CLINICAL MANIFESTATIONS OF METAL ALLERGY

The acute ICD is usually characterized by erythema, blistering, pustules, bleeding, crusting, scaling, and erosions, as well as itching or even pain. Cutaneous lesions in an acute inflammatory CD are predominantly located in areas of contact with a sharp edge (distal spread does not occur) and are usually asymmetric.^23^, ^30^, ^43^

The chronic inflammatory CD is characterized by diffuse or localized lesions with poorly defined erythematous scaly patches and plaques, dry skin, lichenification, and scaling.^24^ In the skin lesions of ACD, there are several different clinical stages, including an erythematous phase with indeterminate erythema or skin edema and *madidans* phase characterized by erosions and moistening.^30^ Next, crusts appear, followed by the final squamous stage, when the stratum corneum repairs itself. Among the general symptoms of ACD, itching is very noticeable.^30^ An acute irritative reaction usually peaks rapidly, within minutes to hours of exposure, and then begins to subside. While in ACD, the time of arousal depends on the characteristics of the sensitizer, the intensity of the exposure, and the degree of sensitivity. Lesions usually appear 24–72 h after exposure to the causative agent and peak at approximately 72–96 h. ACD heals more slowly than ICD and relapses more quickly (within days) after re‐exposure.^22^

**FIGURE 2:**
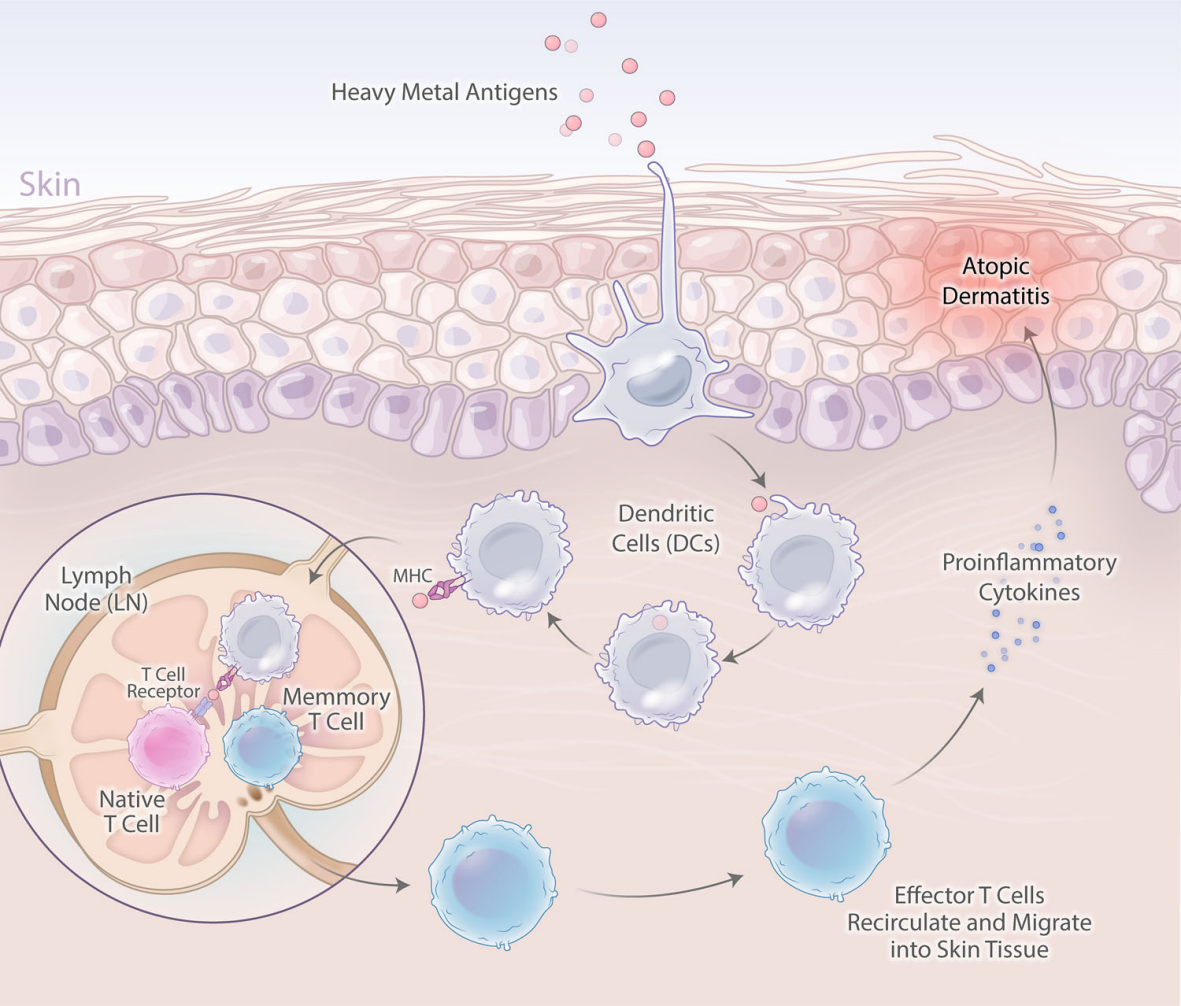
The entry of heavy metals allergens, insect bite, dust mite, pollen, and food into the body and clinical manifestations of metal hypersensitivity. The mechanism of heavy metals and other allergens after exposure to the body shows clinical manifestations of metal hypersensitivity. Dendritic cells subsequently carry contact allergen in the context of protein–MHC complexes to the draining lymph nodes, where activated memory T cells are primed and developed. Recirculating and migrating hapten‐specific T cells after activation produce pro‐inflammatory cytokines cause atopic dermatitis.

Metal ACD is common and is caused by a type IV allergic delayed cellular reaction. In this pathway, repeated or prolonged exposure to certain compounds, such as jewelry, clothing, or implanted devices, sensitizes T cells to activate and induce an immune response at sites of stimulation.^49^, ^50^ Figure 2 indicates the mechanism of heavy metals and other allergens after exposure to the body and reactions and symptoms related to metal allergy.^48^

All three heavy metals Pd, Pt, and Ti have many applications in medical and ornamental fields. Therefore, it is very important to choose these metals and check the allergic effects caused by these metals.

## 5 PALLADIUM ALLERGY

Acute CD as a possible complication of Pd contact can reduce the quality of life of people affected by it. The most common sources of Pd exposure in people are jewelry and dental appliances such as dental crowns, bridges, and inlays. Other sources of exposure to Pd are electronics, chemical catalysts, and Pd released from catalytic converters.^50^, ^51^, ^52^, ^53^, ^54^ Measurements of soil Pd in Germany show that Pd emitted from car exhaust increased Pd in soil along highways 15 times higher than the Pd measured in 1994. Another study shows that Pd and other elements used in catalytic converters have increased in road dust and ambient air 90‐fold higher than their normal amount in the past.^55^, ^56^, ^57^, ^58^, ^59^

Occupational Pd CD is not common. Although occupational Pd CD may occur in some occupations, including those in contact with electronic equipment, dental alloys, sparks, and catalytic converters at work,^50^, ^60^ Pd CD has different manifestations in different people based on exposure time, quality of contact and amount of metal allergen, genetic capacity, and detoxification of the patient.^50^, ^61^

Mucosal, skin rashes and itching at the contact site, urticaria, eczematous and lichenoid histologic changes, linear lichen planus, cheilitis, stomatitis, periodontitis, lip swelling, unusual sensations in the mouth, mouth burning without any other complications and allergic contact granuloma of the sarcoid type (rare) are common manifestations of metal CD.^56^, ^62^, ^63^, ^64^, ^65^ Pd also can cause metal‐induced occupational asthma in refinery workers.^66^ Besides, local skin manifestations, metal contact hypersensitivity can cause systemic symptoms such as dizziness, asthma, lethargy, fever, headache, and arthralgia.^50^, ^60^, ^67^

Another complication that can be associated with metal allergy (particularly Pd and Ni) is ulcerative colitis (UC). It is interesting to know that 60% of UC patients are allergic to at least one metal allergen. This association suggests a possible pathophysiological role of metal ACD in the development of UC. Continuous release of metal ions or metal particles from dental alloys may cause a delayed‐type hyperplasia (DTH) reaction and inflammation in the colonic mucosa. In addition to UC, some other autoimmune diseases, such as rheumatoid arthritis, systemic lupus erythematosus, and Sjogren's syndrome, are associated with metal hypersensitivity.^68^, ^69^

**FIGURE 3:**
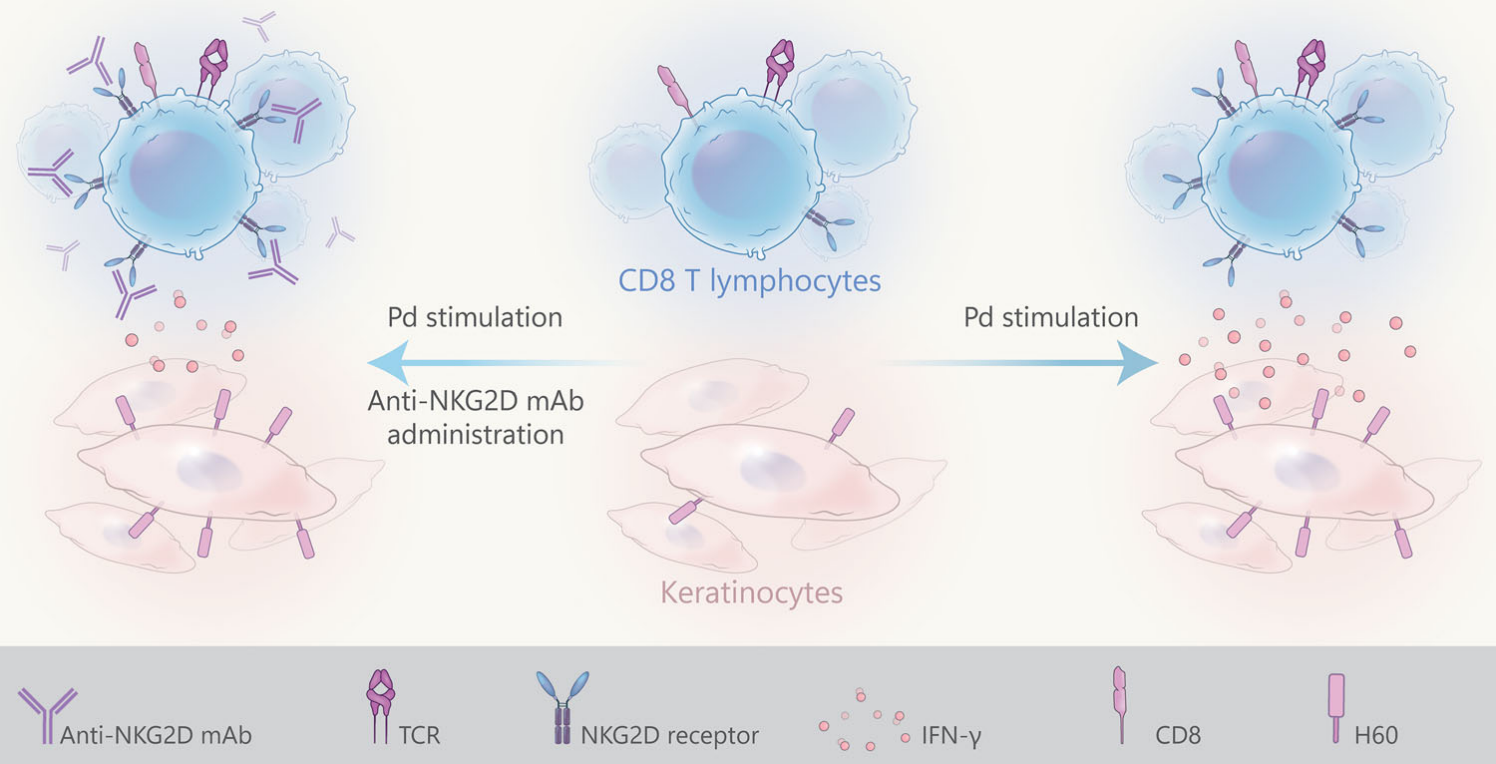
The natural killer group 2D (NKG2D) receptor is expressed on T cells by CD8 cells, natural killer cells, and certain subsets of CD4 T cells and contributes to the development after Pd exposure that increases H60 receptors in skin tissue. H60 acts as a ligand of NKG2D and increases interferon‐gamma (IFN‐γ) secretion, which causes more severe allergic contact dermatitis (ACD) reactions in the ear following Pd administration. The neutralization of NKG2D by in vivo administration of anti‐NKG2D monoclonal antibodies (mAbs) reduces IFN‐γ secretion by CD8 T cells, thereby significantly inhibiting allergic reactions.

Pd CD, like other CD reactions, is a delayed type hypersensitivity reaction or type IV hypersensitivity reaction. In this group of allergic reactions, immune cells, especially T lymphocytes, play the most important role, and acute CD does not occur in the absence of these cells. T cells play a very important role in the development of ACD reactions, such that reactions do not occur in anti‐CD8 monoclonal antibody (mAb) mice. In contrast to anti‐CD8 mAbs mice, ACD occurs which is a more prolonged response in anti‐CD4 mAbs mice^36^, ^50^, ^70^, ^71^ (Figure 3). CD4 (both Th‐1 and Th‐2) and CD8 (the most important) cause ACD with their cytokines (especially IFN‐γ).^36^, ^50^, ^68^, ^71^, ^72^, ^73^ Meanwhile, Kawano et al.^36^ evaluated Pd ACD in nude mice lacking intrathymic T‐cell development. Pd allergy was not observed in nude mice, suggesting that thymus‐derived αβ‐T cells are necessary for the development of Pd ACD. They compared the susceptibility of Pd DTH in CD4‐deficient mice (MHC class 2‐deficient mice) versus CD8‐deficient mice (MHC class 1‐deficient mice). Induction of Pd contact allergy was not observed at all in CD8‐deficient mice but was only prolonged in CD4‐deficient mice.^36^ They also well demonstrated the importance of CD8 in the induction of allergy to Pd. In addition to IFN‐γ, CD8 cells release perforin and granzyme to exert their cytotoxic effects. Despite the cytotoxic effect of perforin and granzyme, they are not essential for the induction of Pd contact allergy. Ear swelling was also observed in perforin‐deficient mice.^36^

During the initial phase (induction phase), Pd ions bind as a hapten to cellular and extracellular matrix proteins, and then the hapten–carrier complexes bind with DC receptors to be subsequently presented to T lymphocytes. At this stage, co‐stimulatory innate immune signals are required to activate DCs and migrate them to skin lymph nodes. In lymph nodes, DCs activate T lymphocytes by presenting haptens to these cells. To achieve maximal activation at this level, T lymphocytes must be stimulated with co‐stimulatory receptors alongside T‐cell receptors. Natural killer group 2D (NKG2D) is a co‐stimulatory receptor that can be seen on normal cells, natural killer (NK) T cells, activated T cells, and memory T cells, but CD4 and naïve T cells do not normally express NKG2D.^36^, ^37^, ^38^

**FIGURE 4:**
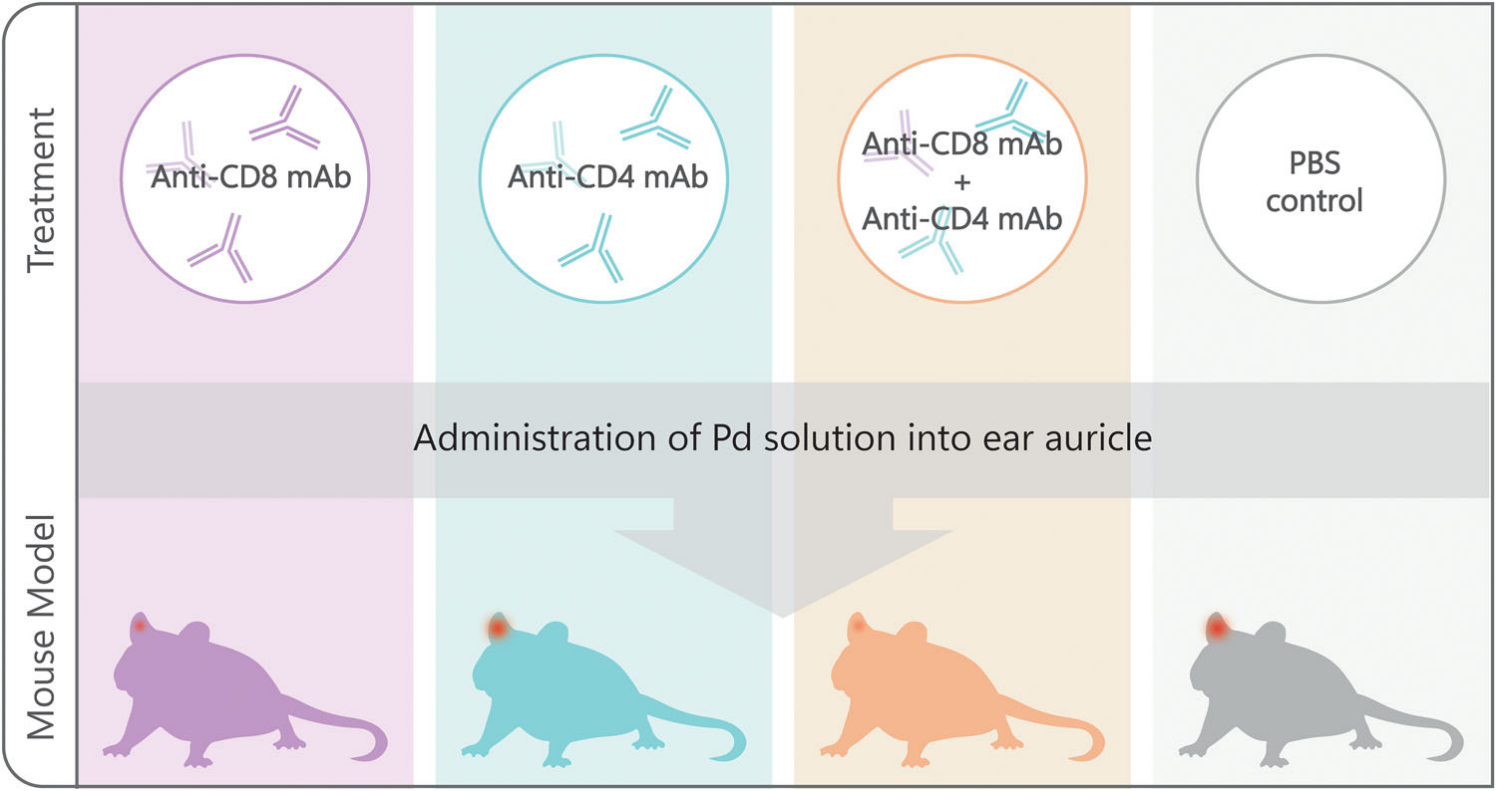
Schematic illustration of the severity of allergy to Pd in the ear of mice treated with CD8+ T cells alone, CD4+ T cells alone, both CD4+ and CD8+ T cells, and rat immunoglobulin G (IgG) as control (phosphate‐buffered saline (PBS)). Ear swelling was observed in control and CD4+ T‐cell‐depleted mice, and ear swelling was significantly inhibited in mice depleted of CD8+ T cells alone or both CD8+ and CD4+ T cells.

NKG2D is an activating receptor expressed by CD8 cells, NK cells, certain groups of CD4 T cells, etc. Histocompatibility 60 (H60) is a family of known ligands for murine NKG2D receptors. NKG2D ligands are stress‐inducible molecules and are increased in inflammatory tissues. REA1 and H60 are known ligands for NKG2D receptors. The interaction of NKG2D and H60 increases the secretion of IFN‐γ, which causes severe ACD reactions. H60 is found significantly in the skin and supports repair of the skin in inflammatory conditions and skin damage. The presence of Pd in ​​the skin, H60, unlike REA1, is significantly increased in the skin. Considering these findings, H60 is the most important NKG2D ligand in the Pd sensitization process. Stimulation of NKG2D receptors on CD8 increases IFN‐γ production by these cells.^36^, ^74^ Down‐regulation or deactivation of NKG2D receptors with its own mAbs reduces the IFN‐γ secretion of CD8 T cells, thereby reducing allergic reactions.^36^, ^75^, ^76^ Likewise, NKG2D receptors were neutralized by the administration of anti‐NKG2D mAb, resulting in significantly reduced ear swelling by Pd^36^ (Figure 4).

An important issue regarding Pd allergy is the cross‐reaction of Ni salts with Pd salts. Hypersensitivity to Pd often occurs with Ni allergy. Durosaro and el‐Azhary found that,^77^ about 94% of people allergic to Pd are also allergic to Ni and in González‐Ruiz et al.^78^ research it was about 90%. However, a small proportion of Pd‐sensitive patients called Pd monosensitive patients do not show allergic reactions to Ni. Pd and Ni are in the same group of the periodic table and are similar to each other. Pd and Ni have the same coordination geometry in the protein‐bound state. Both Pd and Ni prefer to form square planar complexes and bind to similar proteins. These reasons explain why Ni reacts with Pd.^50^, ^79^, ^80^

About 15%−20% of people in Western countries are sensitive to at least one metal allergen.^38^ There are mixed epidemiological data on Pd ACD, and scientists believe that the prevalence of Pd sensitization is underestimated.^63^, ^81^ According to epidemiological data, the prevalence of sensitivity to Pd is 9% in mainland Britain, 9% in Turkey, 8.3% in unselected eczema patients in Austria, 7.4% in Germany, 13% in Israel, and 2% in Northern Ireland. Likewise, Israel and Northern Ireland are unmatched by other countries data. These unmatched statistics may reflect hypersensitivity to Pd in ​​Northern Ireland and Israel or may occur due to different Pd patch testing methods or different errors during patch testing.^64^

Faurschou et al.^50^ tested 10,778 patients with Pd chloride from 1986 to 2008. They found that the mean prevalence of allergy to Pd was 7.8% (<1.0%−19%) in dermatitis patients and 7.4% (1.3%−13.9%) in dental patients. The average prevalence of monosensitivity to Pd was 0.2% (0%−1.6%) in dermatitis patients and 0.5% (0%−7.2%) in dental patients.

In an interesting study by Durosaro et al., only 14% of patients with Ni hypersensitivity had a stronger PT reaction to Pd than to Ni.^77^ Moreover, Rosholm Comstedt et al.^80^ found that the prevalence of Pd allergy has decreased among young women, while it has increased among older women. The prevalence of PdCl_2_ allergy among young women was 16.0% in 1995−1999 and decreased to 9.1% in 2012−2016, but the prevalence of Pd allergy among older women increased from 7.4% to 13.6% from 1999−1995 to 2012−2016. In contrast to women, the prevalence of PdCl_2_ allergy remained almost constant among all age groups in men, although a slight decrease of 2.3% in 1999−1995 and 1.5% in 2012−2016 was observed. Some studies show that the prevalence of Pd allergy has increased over the past 20 years.^80^, ^82^ Pd ACD is more common in females than in males. Two reasons are suggested: the first reason is the hypersensitivity to Ni in women due to Pd allergy is more common in patients with Ni sensitivity. The second reason is that most women use jewelry.^50^ Pd stimulation of human peripheral blood mononuclear cells (PBMCs) obtained from Pd hypersensitivity patients induced lymphocyte proliferation and higher levels of IFN‐γ, IL‐1, TNF‐α, IL‐10, IL‐2, and IL‐4 production compared to the control group.^56^, ^72^, ^83^, ^84^, ^85^

A PT is a validated test to detect sensitivity to Pd. For example, Muris et al.^86^ indicated that sodium tetrachloropalladate (Na₂PdCl₄) is a more sensitive substance for the detection of Pd allergy than PdCl_2_. Furthermore, PBMCs release different cytokines in patients with sensitivity to fossils and isolated Ni–Pd. The enzyme‐linked immunosorbent spot (ELISPOT) assay is an extremely accurate and sensitive method to measure cytokine production of PBMCs in response to Pd stimulation. In addition, the lymphocyte transformation test (LTT) is another promising tool to investigate Pd sensitivity.^84^, ^87^, ^88^, ^89^, ^90^, ^91^, ^92^

Both Pd and Ni can induce a mixed Th‐1 and Th‐2 response in hypersensitive patients, but Th‐2 responses (mainly IL‐5 release) are significantly dominant in Ni stimulation. Therefore, assessment of Th‐2 response can be used to diagnose isolated allergy to Pd from Ni–Pd cross‐reactivity. According to Spiewak et al.^89^ and Czarnobilska et al.,^90^ there is a strong correlation between IL‐5 and predominant Ni hypersensitivity. In an interesting study, Summer et al.^91^ suggested that IL‐5/IL‐8 response ratio could be useful for distinguishing between isolated Pd allergy and predominant Ni allergy.

In another study, Kobayashi et al.^85^ showed that increased IL‐5 was not observed in isolated mice hypersensitive to Pd. They stimulated some isolated Pd‐specific clones with Pd. Following Pd stimulation, Th‐1 cytokines (e.g., IFN‐γ and TNF‐α) and IL‐10 release were detected, but there was no increase in Th‐2 cytokines (e.g., IL‐4 and IL‐5).

PBMCs from Ni‐ and Pd‐negative patch‐tested patients had neither LTT reactivity nor IL‐5 production in response to Ni or Pd stimulation. PBMCs from Pd‐positive and Ni‐negative patch‐tested patients did not release IL‐5 in response to Ni. However, Pd stimulation induced IL‐5 release, but significantly less than IL‐5 release following Ni stimulation in Ni hypersensitive patients. Besides IL‐5, the lymphocyte transportation test or LTT is another test to evaluate metal sensitivity. A positive LTT is associated with IL‐5 production following Ni stimulation, implying Ni sensitivity, but a proliferative response without IL‐5 release following Pd stimulation, implying increased Pd sensitivity, is isolated. Therefore, LTT combined with measurement of IL‐5 release can be a useful tool to distinguish Ni–Pd cross‐reactivity from isolated Pd hypersensitivity.^88^

Pd nanoparticles (PdNPs) and Pd salts have different effects on the body. Since PdNPs can be internalized by cells, it causes direct interaction of PdNPs with intracellular structure. Both Pd salts (Pd salts such as PdCl_2_ and Na_2_PdCl_4_) and PdNPs can induce CD reactions, but PdNPs cause much less allergic reactions than Pd salts.^56^, ^83^ Analysis of PBMCs exposed to PdNPs by transmission electron microscopy shows that PdNPs are endocytosed by PBMCs and form dilated intracellular vacuoles that cause toxic effects on these cells. Swollen cristae in these cells are evidence of mitochondrial damage. PdNPs can induce oxidative and metabolic stress.^56^

Unlike Pd salts and PdNPs, PAPLAL (a mixture of PdNPs and PtNPs) could not regulate IFN‐γ expression; therefore, PAPLAL did not induce Pd allergy. Gene expression analysis showed that PAPLAL can significantly suppress the expression of IFN‐γ, IL‐1β, and TNF‐α genes. Furthermore, PAPLAL can increase the activity of superoxide dismutase, which reduces reactive oxygen species. The pro‐inflammatory cytokines suppress and the antioxidant effect of PAPLAL inhibits skin inflammation and CD, which can benefit from its therapeutic potential in skin diseases such as vitiligo without causing inflammation or an allergic reaction.^83^

### 5.1 Platinum allergy

Exposure to Pt group elements may cause acute toxicity or hypersensitivity with respiratory symptoms, urticaria, and, less frequently, CD. These effects depend on both the exposure intensity and the chemical forms of the metals.^93^, ^94^ Pt is a highly reactive transition metal that partially filled d‐shells^95^ and readily complexes with donor groups in amino acids.^96^ Chlorinated soluble compounds, such as hexachloroplatinic (IV) acid, its ammonium and potassium salts, and potassium and sodium tetrachloroplatinate (II), represent the most dangerous chemical forms. Some of these salts showed strong sensitizing potency in animal models. Using popliteal lymph node assay in mice, Schuppe et al.^97^ showed that hexa and tetrachloroplatinates have the highest sensitizing potency. In the last two decades, the widespread use of catalytic converters has led to the dispersion of Pt emitted from worn surfaces in urban environments, mainly in dust and soil near busy roads, in water, air, and food.^98^, ^99^ About 10% of the emitted Pt is soluble and can be transformed in the environment. Consequently, high levels of Pt group metals, assessed by inductively coupled plasma mass spectrometry, have been found in the urine and plasma of people living in urban environments.^100^ An epicutaneous PT is used to detect metal sensitivity. It is the primary tool for identifying allergens that cause ACD.^101^ Pt is also used in jewelry and dentistry, but mostly as an automotive catalyst. In recent years, there has been an increasing demand for Pt, resulting in increased mining production worldwide and environmental releases of element.^102^ A study of 153 workers in a catalyst manufacturing and recycling plant showed that Pt salts are important allergens in the catalyst industry.^102^ Pt‐induced bronchial asthma has developed in factory workers exposed to Pt‐78 salts.^103^ Moreover, Pt can also cause chemotherapy‐induced sensitivities in Pt‐containing anticancer agents such as cisplatin, carboplatin, oxaliplatin, taxanes (e.g., paclitaxel and docetaxel), L‐asparaginase, epipodophyllotoxins (e.g., teniposide and etoposide), procarbazine, mAbs, and 6‐mercaptopurine.^104^, ^105^, ^106^, ^107^ Immunological mechanisms responsible for clinical sensitivity are IFN‐γ‐producing CD4^+^ and CD8^+^ cells, which are the main factors of sensitivity and are directly responsible for skin manifestations^108^, ^109^ and regulatory T cells secrete IL‐10 and transforming growth factor‐beta (TGF‐β), which modulate the response.^110^, ^111^, ^112^

Furthermore, Pt is widely used in catalytic converters or as a catalyst in chemical reactions, which can be used in the production of heat‐resistant thermometers and electrode production. Pd is also used to produce catalytic converters or as a catalyst for chemical reactions. It is also used in the manufacture of dental instruments and various types of medical prostheses, glucometer electrochemical test strips, electrical instruments, jewelry,^52^, ^113^, ^114^ glass‐making tools, permanent magnets, dental tools, prosthetic manufacturing,^115^, ^116^, ^117^ and anticancer drugs.^12^, ^118^

### 5.2 Titanium allergy

Ti or Ti alloys are widely used in medical devices (e.g., orthopedic surgery, dentistry, and cardiology in pacemaker housings and cardiovascular stents) as an alternative to Ni, chromium, and cobalt, which many patients rely on. Ti is used in patients who have an allergic reaction to Ni, chromium, and cobalt elements.^119^, ^120^, ^121^, ^122^, ^123^ Furthermore, Ti is used in jewelry and as a white pigment (TiO_2_) in personal health care products such as toothpaste, cosmetics, and sunscreen,^124^, ^125^ and therefore daily human exposure to Ti is increasing.^126^, ^127^ Ti is combined with a variety of other elements to produce strong lightweight alloys that provide corrosion resistance and exhibit very high strength/density ratios.^128^ Due to the excellent biocompatibility of Ti, Ti and commercially pure Ti alloys have been widely used as alternatives to other metals in invasive medicine, surgery, and dentistry during the last three decades.^129^ However, Ti‐based implants can release particles and ions into surrounding tissues and body fluids.^130^, ^131^, ^132^ In their ionic form, metals can bind to native proteins to create haptic antigens or can cause degranulation of mast cells and basophils and are capable of inducing type I or type IV sensitized reactions.^133^, ^134^ TiO_2_ NPs can enhance Th2‐dominant immune response with high levels of specific immunoglobulin E (IgE) and IgG1 in serum. It activates the NLR pyrin domain‐containing 3 (Nlrp3) inflammasome, which is involved in inflammation and other immune responses, leading to IL‐1β release.^135^, ^136^ One of the side effects of Ti in cosmetic products is the potential to penetrate through the stratum corneum, reach the living layers of the skin, and eventually cause sensitivity or other side effects.^137^ Although Ti allergy has a low prevalence rate, for patients with a previous history of allergies, metal sensitivity evaluation, and allergy testing may be recommended prior to placement of a permanent implant to prevent implant failure due to an allergic reaction.^138^ Hosoki et al.^139^ demonstrated an allergic reaction to Ti in dental and orthopedic implants in the case report. Disruption of osteosynthesis was reported in one case with a Ti orthopedic implant in 2006 in Germany.^140^ Systemic disease can occur due to Ti toxicity. Berglund and Carlmar^141^ found that Ti toxicity can cause nail yellowing, bronchial obstruction, lymphedema, postnasal drip, and sinusitis associated with cough, which they termed yellow nail syndrome. Wang et al.^142^ reported that acute Ti poisoning caused damage in the liver (aqueous degeneration around the central vein in the liver and patchy necrosis of hepatocytes) and kidneys (blood urea nitrogen (BUN) level increased with renal pathological changes) after high‐dose (5 g/kg body) oral administration of different sizes of TiO_2_ particles (25, 80, and 155 nm).

On the other hand, in cardiovascular procedures, metal sensitivity has caused the failure of intracoronary stents, as evidenced by repeated in‐stent restenosis.^143^ Plastic and dental surgery involving Ti or other metal implants have also shown adverse allergic reactions and subsequent complications from implants.^144^ Hypersensitivity to various metals is a frequently emerging problem due to significant side effects.^145^ Pd is a widely used component in most cast dental restorations and amalgams (dental alloys may contain up to 10% Pd).^52^, ^146^, ^147^, ^148^ Over the past decade, numerous reports of sensitization to Pd as an allergen have been documented to contact allergy of the oral mucosa: cheilitis, stomatitis, burning mouth, lichenoid reactions, oral and facial granulomatosis, or swelling of the lips and cheeks, dizziness, asthma, and chronic urticaria.^65^, ^148^, ^149^, ^150^ Due to the increase in the use of Pd and as a result of exposure to this metal, the level of sensitivity has increased.^63^ Metallic Pt is used in jewelry, photography, dentistry, and in the chemical and electrical industries and can also be used as a catalyst.^151^ Occupational exposure occurs during Pt mining and processing. However, the most common current occupational exposure to soluble Pt compounds is through Pt refining and catalyst production.^102^ Occupational exposure to soluble Pt has been associated with some adverse skin reactions (dermatitis, eczema, and urticaria) and respiratory health effects (asthma, rhinitis, and dyspnea).^152^ An epicutaneous PT is a tool used to identify the factors that cause ACD. It is a scientific method of investigation, with internationally defined rules and well‐established foundations. The performance of the PT produces in a controlled manner the arousal phase of ACD and thus determines the causative agent of this dermatitis.^153^ Apart from the skin PT, blood‐based tests such as the lymphocyte conversion test or the leukocyte migration inhibition test can be used to determine delayed‐type hypersensitivity to metals.^154^ In addition, flow cytometry has been proposed as a method to evaluate sensitivity to metal implants.^155^ The increasing frequency of metal allergies in the general population raises the potential need for screening surgical patients to prevent potential allergy‐related complications. The American Contact Dermatitis Society recommends skin patch testing before device implantation for patients with a clear history of metal reactions.^156^ It can also affect the regulation of metal content in consumer products. Nevertheless, the avoidance of allergens (e.g., switching to alternative materials such as ceramics for implants) is the most effective prevention for triggering metal allergies.^157^

## 6 THERAPEUTIC APPROACHES

Treatment of Pd, Pt, and Ti ACD, like any other type of dermatitis, depends on the extent of skin involvement, its severity, and severity. To manage ACD, the offending agent should first be determined based on patient history and diagnostic tests such as PT and LTT. Patch testing is a gold standard test for allergen determination, but some limitations make it difficult to perform.^158^, ^159^, ^160^, ^161^ Allergen avoidance is the most important part of managing ACD, but it is not always easy. Educating the patient and familiarizing patients with proper ways to avoid allergens is one of the duties of the medical team. Patients should be taught to use allergen‐free products and appropriate clothing such as gloves, especially in the workplace.^21^, ^158^, ^162^, ^163^, ^164^

**FIGURE 5:**
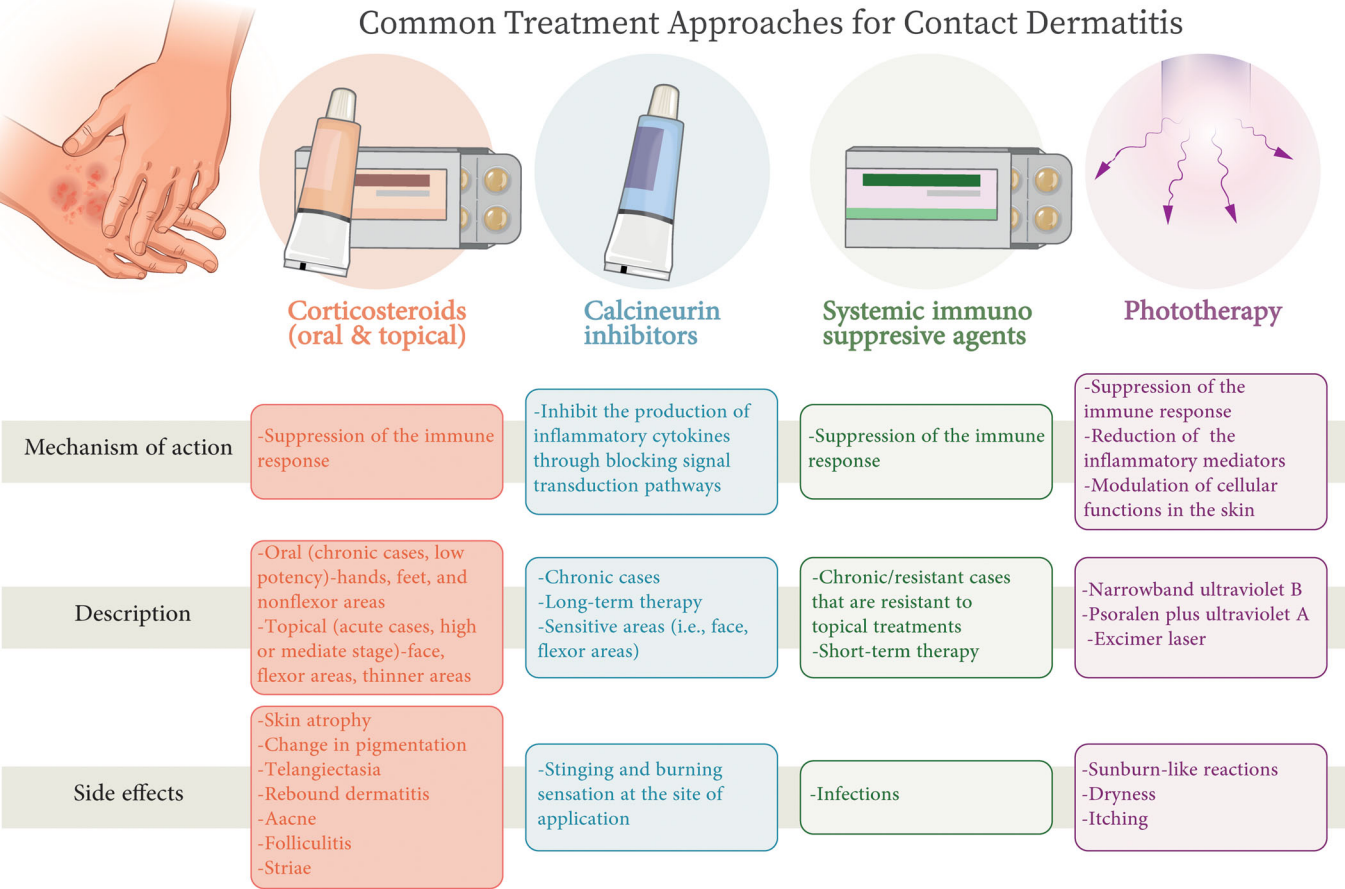
Common treatment approaches for contact dermatitis. Mechanisms of action, description, and side effects were proposed for corticosteroids (oral and optical), calcineurin inhibitors, systemic immunoresponsive agents, and phototherapy.

The use of barrier creams, ointments, and moisturizing creams is highly recommended. These agents have protective effects on the skin barrier and reduce the risk of ICD (ICD is associated with a higher risk of ACD due to its immunomodulatory effects and increased skin permeability). Barrier creams (chelating and non‐chelating barrier creams) may reduce penetration of the offending substance through the skin. The effectiveness of chelating barrier creams in blocking Pd, Pt, and Ti ions is unclear. Wöhrl et al. demonstrated the protective effects of DTPA in oil–water emulsion in patients with Ni allergy. However, this protective effect was not observed with palladium chloride.^158^, ^165^, ^166^, ^167^, ^168^, ^169^ Allergen avoidance is a key factor in the management of ACD, so drug treatments such as corticosteroids, calcineurin inhibitors, immunosuppressive agents, phototherapy, antihistamines, etc., are often used with the goal of faster recovery. Figure 5 indicates common treatment approaches for CD, including mechanisms of action, description, and side effects for corticosteroids (oral and optical), calcineurin inhibitors, systemic immunoresponsive agents, and phototherapy.

### 6.1 Corticosteroids

Topical corticosteroids are the most common treatment in cases of ACD. Corticosteroids have an inhibitory effect on stratum corneum repair, although their effectiveness in the treatment of ACD has been strongly confirmed.^170^, ^171^ The use of moderate‐ to high‐potency topical corticosteroids (e.g., clobetasol 0.05% or triamcinolone 0.1%) is recommended in cases of acute, localized ACD in the hands, feet, and nonflexor areas. In cases of acute ACD involving the face, flexor areas, and areas with thinner skin, low‐potency topical corticosteroids such as desonide ointment are recommended to reduce the side effects of corticosteroids.^21^, ^158^, ^159^, ^160^, ^172^, ^173^, ^174^, ^175^ Topical corticosteroid overuse may cause side effects such as skin atrophy, change in pigmentation, telangiectasia, rebound dermatitis, acne, folliculitis, striae, etc. These side effects (especially skin atrophy) are more common in the face and intertriginal areas.^8^, ^158^, ^159^, ^160^ Oral corticosteroids are preferred in ACD involving more than 20% of body surface area, chronic ACD, and debilitating ACD. Rapid discontinuation of corticosteroids may cause relapsing dermatitis, so tapering oral corticosteroids over 2–3 weeks is recommended.^8^, ^27^, ^159^, ^160^, ^175^, ^176^

### 6.2 Topical calcineurin inhibitors

Since topical calcineurin inhibitors do not cause skin atrophy (unlike corticosteroids), topical calcineurin inhibitors are recommended instead of topical corticosteroids in chronic ACD, ACD requiring long‐term use of topical therapy, and ACDs that involve sensitive areas such as the face and flexural areas.^8^, ^158^, ^168^, ^177^, ^178^, ^179^, ^180^, ^181^ Calcineurin inhibitors such as tacrolimus and pimecrolimus block signal transduction pathways, thereby inhibiting the production of inflammatory cytokines.^160^, ^182^ The most common side effects of tacrolimus are stinging and burning sensation at the site of application.^160^ Pimecrolimus is another calcineurin inhibitor that is three times more potent than tacrolimus.^160^, ^183^ Pimecrolimus 0.6% cream was effective in the treatment of Ni‐induced ACD.^184^ But Mose et al.^185^ did not observe any significant effect of pimecrolimus cream 1% on ACD. The most common side effect of topical pimecrolimus was a burning sensation at the application site, which lasts for about 3 days in most patients.^160^

### 6.3 Systemic immunosuppressive agents

Systemic immunosuppressive agents are sometimes used in the treatment of severe or refractory cases of CD, particularly when other treatment options have been insufficient. However, their use is generally reserved for specific situations and managed under the supervision of a dermatologist or an allergist. Systemic immunosuppressive agents such as cyclosporine, azathioprine, or mycophenolate can rarely be used in chronic and resistant ACD.^186^, ^187^, ^188^ There are some points to know about the treatment of CD with systemic immunosuppressive agents. Systemic immunosuppressive agents may be considered in cases of severe CD that are resistant to topical treatments or in those where the condition significantly affects a person's quality of life. Different systemic immunosuppressive agents can be prescribed depending on the severity of the condition, the patient's overall health, and individual factors. Commonly prescribed agents may include oral corticosteroids (e.g., prednisone), immunomodulatory drugs (e.g., cyclosporine, azathioprine), or biologic therapies (e.g., dupilumab). The choice of medication depends on various factors and requires expert evaluation and monitoring. Systemic immunosuppressive agents are typically prescribed for a limited period, typically for short‐term use or until the symptoms are adequately under control.^187^, ^189^, ^190^, ^191^, ^192^ Long‐term use of these agents can increase the risk of side effects, complications, and infections. The treatment duration is determined based on individual response and the assessment of risks versus benefits. It is important to note that the use of systemic immunosuppressive agents for CD is considered a specialized treatment approach and should only be initiated and managed by healthcare professionals experienced in their use. The decision to use these agents requires a careful evaluation of the specific situation and a thorough consideration of the potential risks and benefits for each individual patient.

### 6.4 Phototherapy

Phototherapy, also known as light therapy, can be an effective treatment option for certain types of CD. It involves exposing the affected skin to specific wavelengths of light in order to alleviate symptoms and promote healing. There are some key points about phototherapy for CD, including narrowband ultraviolet B (NB‐UVB) therapy, psoralen plus ultraviolet A (PUVA) therapy, and excimer laser.^193^, ^194^, ^195^ Phototherapy is believed to work by several mechanisms, including suppression of the immune response, reduction of inflammatory mediators, and modulation of cellular functions in the skin. The exact mechanisms and effects can vary depending on the type of phototherapy used. Phototherapy involves exposing the affected skin to ultraviolet (UV) light to reduce inflammation and alleviate symptoms.^196^, ^197^, ^198^, ^199^ Phototherapy is generally considered safe and well tolerated, but it does have potential side effects, such as sunburn‐like reactions, dryness, and itching. It is important to undergo phototherapy under the supervision of a healthcare professional who can monitor your progress and adjust the treatment as needed. It is important to discuss the potential benefits and risks of phototherapy with a healthcare professional before undergoing treatment. UV radiation has an innate immunosuppressive effect. Topical PUVA has more cytotoxic effects than UVB, so it is suggested to start phototherapy with UVB and continue treatment with PUVA if UVB is not effective.^160^, ^200^

### 6.5 Antihistamines

Antihistamines cannot significantly reduce pruritus in ACD patients.^175^ However, the sedative effect of hypnotic antihistamines such as diphenhydramine and hydroxyzine may help patients.^8^, ^159^ Promising new therapies such as anti‐TNF‐α mAbs, phosphodiesterase (PDE4) inhibitors, and topical nicotinamide adenine dinucleotide (NADH) may help patients in the future.^160^, ^201^ Medical metal implants can cause allergic reactions that may cause implant failure, which can be recognized by manifestations such as dermatitis, impaired wound healing, effusion, pain, and loosening.^202^, ^203^, ^204^ Nevertheless, metal implant allergic reactions do not always result in implant failure or disabling outcomes.^205^, ^206^ Due to the risk of systemic contact dermatitis (SCD) and implant failure, patients' history of metal sensitivity should be checked before implantation. If the patient's history of metal sensitivity is positive, PT should be performed to provide patients with an allergen‐free implant.^161^, ^202^ However, patients with a history of negative metal sensitivity may have positive PT results, many experts believe that PT is not necessary in patients without a history of metal sensitivity.^161^, ^202^, ^207^, ^208^, ^209^, ^210^, ^211^

If metal ACD symptoms appear after PT implantation, it is mandatory and specialists must confirm hypersensitivity reactions based on defined criteria. In cases of ACD to metal implants, it is necessary to minimize exposure to these metals in the environment and food. Implant removal is the last choice in patients whose medical treatment (such as corticosteroids) is not effective.^161^, ^202^, ^212^ Removal of the implant without replacement, replacement of the primary implant with a non‐allergic implant, and metal coating with polytetrafluoroethylene are possible options for patients and their surgeons.^161^, ^209^

## 7 FUTURE PRESPECTIVES

The future direction for the diagnosis and treatment of ACD to heavy metals is likely to involve advancements in both diagnostic techniques and therapeutic options. Here are some potential areas of development:

1. *Improved diagnostic tests*: Currently, patch testing is the gold standard for diagnosing ACD. However, the process can be time‐consuming and may not always accurately identify the specific heavy metal allergen responsible for the reaction. Future developments may focus on enhancing the accuracy and efficiency of patch testing, such as through the use of novel allergen panels or improved detection methods.
2. *Molecular diagnostics*: Advancements in molecular diagnostics, such as DNA microarray technology or gene expression profiling, could potentially aid in identifying specific genetic markers associated with heavy metal allergies. This could help in predicting an individual's susceptibility to allergic reactions and enable targeted preventive measures.
3. *Novel biomarkers*: Exploration of novel biomarkers specific to metal‐induced ACD may aid in the diagnosis and assessment of disease severity. Identifying specific biomarkers associated with metal exposure and immune response can enhance diagnostic accuracy.
4. *Personalized medicine*: With a better understanding of the genetic and immunological factors involved in heavy metal allergies, personalized medicine approaches may emerge. Tailoring treatment plans based on an individual's specific genetic profile and immune response could lead to more effective and targeted therapies, minimizing adverse effects and optimizing outcomes.
5. *Novel therapeutic options*: Currently, treatment for ACD to heavy metals mainly involves avoiding exposure to the allergen, using topical corticosteroids, and managing symptoms. In the future, there may be advancements in therapeutic options, such as the development of targeted immunomodulatory drugs or biologics that can specifically inhibit the allergic response to heavy metals.
6. *Targeted therapies*: As our understanding of the underlying mechanisms of metal allergies improves, it may be possible to develop targeted therapies that specifically address the immune response triggered by heavy metals. This could involve the development of drugs or biologics that modulate key immune pathways involved in ACD.
7. *Immunomodulatory treatments*: Immunomodulatory therapies, such as immune checkpoint inhibitors or cytokine‐targeting agents, may be explored for their potential to regulate the exaggerated immune response seen in ACD.
8. *Nanotechnology‐based solutions*: Nanotechnology holds promise in various fields, including medicine. In the context of ACD to heavy metals, nanoparticles could potentially be utilized for targeted drug delivery, enhancing the efficacy of treatments while minimizing systemic side effects.
9. *Education and prevention*: Increasing public awareness about heavy metal allergies and promoting preventive measures can play a crucial role in reducing the incidence and severity of ACD. Future efforts may focus on educating individuals about potential sources of heavy metals, proper handling techniques, and the importance of early diagnosis and treatment.
10. *Occupational safety*: Stronger occupational safety regulations, improved guidelines, and stricter metal exposure monitoring in high‐risk professions could help reduce the incidence of metal‐induced ACD.

Overall, the future direction for the diagnosis and treatment of ACD to heavy metals is likely to involve a multidisciplinary approach, combining advancements in diagnostics, therapeutics, personalized medicine, and preventive strategies. These developments aim to improve patient outcomes, enhance quality of life, and reduce the burden of heavy metal allergies.

## 8 CONCLUDING REMARKS

ACD is a common occupational dermatosis associated with significant disease burden in the workforce.

Heavy metals can act as allergens in causing CD. According to extensive studies on allergic reactions caused by heavy metals including Pd, Pt, and Ti, these metals are found to be remarkably common. A deeper understanding of the mechanisms and molecular pathways of these allergens can enlighten our knowledge about a variety of allergies, including ACD. Improvements in population health care and treatment methods require the design of implant biomaterials with minimal or no harmful effects on the host tissue. Metal manipulation and hyposensitive coating are common options for reducing the sensitivity of metals. The most important part of ACD and ICD treatment is avoiding triggers, including heavy metal allergens and other triggers. Clinicians should consider reporting metal/jewelry sensitivity for more accurate treatment of skin allergies. Moreover, dedicated guidelines can be used to recognize the ingredients of medical and industrial products under international standards for people sensitive to metal hypersensitivity and to prevent public concern. In occupational cases, patients may need to change the work environment, use protective equipment, and/or change jobs. To minimize occupational CD caused by metals, rules and protective equipment for metal refinery workers should be considered. Before implanting dental and orthopedic implants, patch testing can help reduce the adverse side effects of metal sensitivity. On the other hand, the development of non‐invasive, sensitive, and specific tests based on cytokine response in in vitro or in vivo models could be very useful in supplementing PT, which could improve the clinical diagnosis of metal allergic. Furthermore, the identification of genes and patterns of the immune system that are involved in the pathogenesis of metal‐induced dermatitis can help patients before medical procedures. New treatments also focus on desensitization without the side effects of corticosteroids and immunosuppressive drugs. We hope that this content can be a suitable and comprehensive quality guide for a deeper and more expressive understanding of this matter.

## AUTHOR CONTRIBUTIONS

*Conceptualization, validation, writing—original draft, and writing—review and editing*: A.S. and A.P. *Formal analysis, conceptualization, visualization, validation, writing—original draft, writing—review and editing, and project administration*: I.Z. *Conceptualization, visualization, and validation*: S.Z.N. *Conceptualization, validation, and supervision*: S.Ch., A.N., and E.M. All authors have read and approved the final manuscript.

## CONFLICT OF INTEREST STATEMENT

The authors declare that they have no conflicts of interest.

## ETHICS STATEMENT

Not applicable.

## Data Availability

Not applicable.
